# Editorial: Chronic Pain and Health Disparities in Older Adults With Complex Needs

**DOI:** 10.3389/fpain.2022.941476

**Published:** 2022-06-28

**Authors:** Maile Young Karris, Margaret Danilovich

**Affiliations:** ^1^Department of Medicine, University of California, San Diego, San Diego, CA, United States; ^2^CJE Senior Life, Chicago, IL, United States

**Keywords:** health disparities, pain, older adults, vulnerable, special populations

Decades of research have demonstrated that disparities in pain management access and outcomes exist (1–4). These disparities are present in all health care settings (i.e., primary care, the emergency room, post-operative, and palliative) across all types of pain (i.e., cancer, neuropathic, acute, chronic) (5–9). Despite this overwhelming evidence, little progress has occurred because barriers to equitable healthcare exist at multiple levels from the individual level to healthcare providers, healthcare systems, and local governments (1, 3, 10–17). A person with pain may not believe that non-pharmacologic therapies such as behavioral therapy will work and thus may not follow up or engage in these proven treatments, especially if there is a share of cost (19–21, 23). The implicit biases (race, gender etc.) of healthcare providers contribute to differences in pain intervention access and medication doses for patients who are Black, Indigenous, People of Color (BIPOC), and women (1, 2). Despite extensive studies that non-pharmacologic therapies such as massage and physical therapy are helpful for pain management, these services are inconsistently covered by health insurance that limits access to people who can pay out of pocket (22). The complicated intertwining of pain management and the opioid epidemic is leading to shortages of providers willing to manage pain (18).

Gender and race disparities persist in later-life pain experiences and pain management that impact patients' quality of life, mental health, function, and cognition (32–34). Pain management in older adults is further complicated by normal age-related changes in pharmacokinetics and pharmacodynamics further limiting pain medication options including non-opioid therapies such as non-steroidal anti-inflammatory drugs and muscle relaxants due to increased risks of side-effects (such as gastrointestinal bleed and cognitive function) and polypharmacy (24–27). Thus, non-pharmacologic pain treatments are central to the management of pain in older adults, but common barriers including awareness, appeal and approach persist (28–31, 35). Concerns about the side effects of pain medicine (including opioids) also result in the under treatment of pain in older adults, further contributing to age-associated pain disparities (36–39).

This Research Topic aims to promote work to enhance our understanding of the disparities that impact vulnerable older adults with chronic pain and influence innovation and policy that addresses disparities to enhance equity. Allen-Watts et al. reported a secondary analysis of the study Examining Racial and SocioEconomic Disparities (ERASED), which focused on individuals with chronic low back pain. Significant associations emerged between race and the use of pharmacologic therapies for pain with Non-Hispanic Whites (NHW) being twice as likely to take one or more medications for pain than Non-Hispanic Blacks (NHB). Opioid use was similar, but NHW were more likely than NHB to utilize antidepressants and non-steroidal anti-inflammatory drugs. Because the national area deprivation index (NADI) was significantly greater in NHB, the authors hypothesized that access to medications and care contribute to differences in pharmacologic treatments. In this study, age did not impact the use of one or more pharmacologic treatments. However, older age and female gender did impact the utilization of primary and tertiary care for pain. For every decade of increased age, utilization of primary or tertiary care for pain increased the odds by 30%. The authors hypothesized that age-associated access to MediCare and more frequent utilization of healthcare providers due to additional medical comorbidities contributed to these differences.

The next article by Milani et al. focused on describing the relationship between multi-morbidity and pain in community-dwelling Mexican Americans aged 80 years+. Of the 841 participants in the Hispanic Established Populations for the Epidemiologic Study of the Elderly, 77.3% reported multimorbidity and 64.1% were female. Participants with multimorbidity had greater odds of pain on weight bearing (odds ratio or OR = 2.27, 95% confidence interval or CI: 1.74, 2.95) pain that limited their daily activities (OR = 2.12, 95% CI: 1.61, 2.78). High depressive symptoms were associated with higher odds of pain on weight bearing (OR = 1.69, 95% CI: 1.35, 2.12) and pain that limits daily activities (OR = 1.88, 95% CI: 1.50, 2.35). Higher cognition was associated with lower odds of pain that limits daily activities (OR = 0.98, 95% CI: 1.50, 2.35). The authors suggest that the association of multimorbidity and chronic pain in older adults complicates pain management and, ultimately, function and quality of life.

You et al. evaluated the age-associated differences of high impact chronic pain (HICP) in a sample (*N* = 133) of mostly female (61.4%), married (62.7%), and highly educated persons (94% with some college or more). Using the graded chronic pain scale-revised (GCPS-R) 69.9% of the sample reported HICP. Age did not impact pain scores but did affect function in different areas. Both younger and older adults stated that pain commonly impacted basic physical activities and the instrumental activities of daily living. However, younger people were more likely to report pain impact on work (but were also more likely to be working 87.3 vs. 10.7%). Older adults reported the impact of pain on participation in social activities (fun) more commonly than younger persons, outlining its contribution to cognitive burden in activity planning, and interference with intimate relationships. The authors discussed that their findings could inform discussions about the impact of pain on function in different domains based on age.

These contributions explore chronic pain across a diverse group of older adults and ultimately encourage readers to (1) consider the potential ability of healthcare policy to combat the social disparities of health and its outcomes, (2) challenge a “one-size fits all” approach to pain management of older adults by demonstrating that chronic pain is often accompanied by multimorbidity that may modify the outcomes of certain pain treatments, and (3) understand that pain may differentially impact older adults specifically by limiting social interactions and sex. To truly reach equity in the management of pain in vulnerable older adults ongoing advancements in healthcare policy, development of person-centered or precision medicine approaches to care and ongoing engagement of healthcare providers to better understand what matters most to older adults in pain are needed.

## Author Contributions

All authors listed have made a substantial, direct, and intellectual contribution to the work and approved it for publication.

## Funding

MK's effort as an editor is in part supported by the National Institutes of Health (P30 AI036214 and R03 AG060183).

## Author Disclaimer

The content is solely the responsibility of the authors and does not necessarily represent the official views of the National Institutes of Health.

## Conflict of Interest

MD was employed by CJE Senior Life. The remaining author declares that the research was conducted in the absence of any commercial or financial relationships that could be construed as a potential conflict of interest.

## Publisher's Note

All claims expressed in this article are solely those of the authors and do not necessarily represent those of their affiliated organizations, or those of the publisher, the editors and the reviewers. Any product that may be evaluated in this article, or claim that may be made by its manufacturer, is not guaranteed or endorsed by the publisher.
